# Structures of distantly related interacting protein homologs are less divergent than non‐interacting homologs

**DOI:** 10.1002/2211-5463.13492

**Published:** 2022-10-17

**Authors:** Nagarajan Naveenkumar, Vasam Manjveekar Prabantu, Sneha Vishwanath, Ramanathan Sowdhamini, Narayanaswamy Srinivasan

**Affiliations:** ^1^ Molecular Biophysics Unit Indian Institute of Science Bangalore India; ^2^ National Centre for Biological Sciences Tata Institute of Fundamental Research Bangalore India

**Keywords:** asymmetry, interacting homologs, protein–protein interaction, sequence identity, structural similarity

## Abstract

Homologous proteins can display high structural variation due to evolutionary divergence at low sequence identity. This classical inverse relationship between sequence identity and structural similarity, established many years ago, has remained true between homologous proteins of known structure over time. However, a large number of heteromeric proteins also exist in the structural data bank, where the interacting subunits belong to the same fold and maintain low sequence identity between themselves. It is not clear if there is any selection pressure to deviate from the inverse sequence–structure relationship for such interacting distant homologs, in comparison to pairs of homologs which are not known to interact. We examined 12,824 fold pairs of interacting homologs of known structure, which includes both heteromers and multi‐domain proteins. These were compared with monomeric proteins, resulting in 26,082 fold pairs as a dataset of non‐interacting homologous systems. Interacting homologs were found to retain higher structural similarity than non‐interacting homologs at diminishing sequence identity in a statistically significant manner. Interacting homologs are more similar in their 3D structures than non‐interacting homologs and have a preference towards symmetric association. There appears to be a structural constraint between remote homologs due to this commitment.

## Graphical abstract

Interacting homologous proteins and homologous proteins which are not known to interact are compared and analyzed for their sequence–structure relationship. Mannose‐specific agglutinin exists as a heteromer (ALLSA I lectin & ALLSA II lectin) which is compared with two non‐interacting homologous pair ALLSA I lectin and Curculin1 protein. We found that interacting protein homologs prefer symmetric associations and their subunits are structurally more similar than the homologous proteins that are not known to interact.

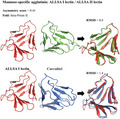

**Abbreviations**
PDB: protein data bank
RMSD: root mean square deviation
SDM: structural distance metric

Protein evolution is characterized by sequence and structural differences between homologous proteins. Relationship between sequence similarity and structural divergence between homologous proteins was first described by Chothia and Lesk [1]. They analyzed 32 homologous pairs of proteins of known structure and derived an inverse relationship between sequence identity and root mean square deviation (RMSD). This landmark work made a major impact especially in the areas of protein structure prediction and modeling.

Apart from the relationship between sequence similarity and structural divergence, it has been shown that the structural homologs can share low sequence identity [2, 3]. In their analysis, of the 32 homologous pairs, there are six protein pairs with low sequence identity (less than 35%) and low structural difference (RMSD value less than 1.5 Å) [1]. This observation indicates that some homologous proteins retain highly similar 3D structures even at low sequence identity. With the availability of many protein structures, the homology between proteins is more confidently ascertained as a result of structural similarity and by consideration of their functions [4]. The classic work by Chothia and Lesk has been revisited by many further works [5, 6] which have laid the foundation to study all areas of protein structure prediction, inverse folding and many other. They have contributed to improving our understanding of evolution of protein structures [7, 8, 9, 10].

These previous analyses were largely confined to homologous proteins which do not interact with each other, i.e., homologous proteins from different organisms that are not known to interact with each other, or non‐interacting paralogs were considered. In this study, we have performed analysis for a special class of homologous protein systems, where the homologous protein modules are known to interact with each other as heteromers and interacting homologous domains in multi‐domain proteins. Interacting protein systems are known to co‐evolve in order to maintain their interactions [11, 12, 13]. We aim to explore if co‐evolution of interacting homologs influence extent of similarity in their three dimensional structures [14, 15]. We investigated this aspect using a dataset of 3D structures of complexes of interacting remote homologs and compared it with non‐interacting homologs of known 3D structures from PDB [16].

## Materials and methods

Three different datasets, corresponding to non‐interacting homologs, heterodimers with homologous subunits and domain repeats of multi‐domain proteins were created for our analysis. All the three datasets constitute proteins of known crystal structure determined at 3 Å or better resolution, and are non‐redundant at 95% sequence identity (i.e., no two entries in the dataset have more than 95% sequence identity). These are discussed further below.

### Dataset of non‐interacting homologs

We considered homologous protein structures that exist as monomer in the asymmetric or biological unit as deposited in the Protein Data Bank; these monomeric homologs are from the same organism as well as from different organisms and are not known to interact with each other. The evolutionary relatedness between the monomers are identified based on their placement in the structural hierarchy at the fold level by SCOPe database.

The non‐interacting homologs dataset used in the current work consists of 26,082 pairs (provided in File S1) of homologs corresponding to 2126 monomeric proteins of known structure from PDB.

### Dataset of interacting homologs

#### Heterodimers

We obtained 3D structures of protein assemblies from PDB and considered all pairs of interacting subunits. In our analysis, we refer them as heterodimers although they could be a part of multimeric protein assembly. We chose those pairs of subunits which are interacting and are likely evolutionarily related. The interacting protein subunits were ensured to correspond to the same structural fold by SCOPe definition [17]. Protein–protein interface residues are identified using Protein Interaction Calculator [18]. The subunits are considered to be interacting only, when the geometric mean of number of interface residues from both subunits are greater than five. This dataset consists of 12,639 interacting sub‐units or heterodimers (provided in File S2) with interacting homologous subunits corresponding to 1875 PDB entries.

#### Domain repeats of multi‐domain proteins

The third dataset consists of homologous domains which are evolutionarily related and interacting in a muti‐domain protein. These are two‐domain proteins from PDB, where both the domains in the protein chain correspond to same structural fold by SCOPe definition. Hence, these are PDB entries where two structurally similar repeat‐domains with significant inter‐domain interactions. Spatial interaction between the two domains was confirmed using the same criteria as used for heterodimers. This dataset finally composed of 185 domain pairs (provided in File S3) corresponding to 185 PDB entries.

Both the datasets described above correspond to datasets of interacting proteins of known structure.

### Structural similarity measures

Structural superposition and structure‐based sequence identity have been carried out using TM‐align algorithm [19], whereas the structural difference between two superimposed protein structures has been measured by Structural Distance Metric (SDM) [20]. SRMS is a converted similarity measure of RMSD and a convenient representation that scales between 0 and 1. It is calculated as  1 − RMSD/5 Å. Pairwise fractional topological equivalence (PFTE) is the ratio of the number of equivalences to the total number of residues in the smaller protein. pymol has been used for graphics visualization and presentation of 3D structures of proteins in the analysis [21].

$$\mathrm{SDM}=-100\log\left[\left(w_{1}*\text{SRMS}\right)+\left(w_{2}*\text{PFTE}\right)\right]$$

SRMS=1−r.m.s.dinÅ/5Å

$$\text{PFTE}=\frac{\mathrm{No}.\text{of equivalent}\;\mathrm{C\alpha}\;\text{atoms}}{\mathrm{No}\;.\;\text{of residues in the smallest protein}}$$

$$w_{1}=\frac{\left[\left(1-\text{SRMS}\right)+\left(1-\text{PFTE}\right)\right]}{2}\;w_{2}=\frac{\left(\text{SRMS}+\text{PFTE}\right)}{2}$$

The structural asymmetry in the heterodimers, for topologically equivalent C‐alpha atoms, was calculated using a method proposed for homodimers earlier [22].

### Statistical test and plotting

All the plotting, curve fitting, and statistical tests were performed using numpy, scipy, and pylab packages from python(Python software foundation, Beaverton, OR, USA).

## Results and discussion

**Fig. 1:**
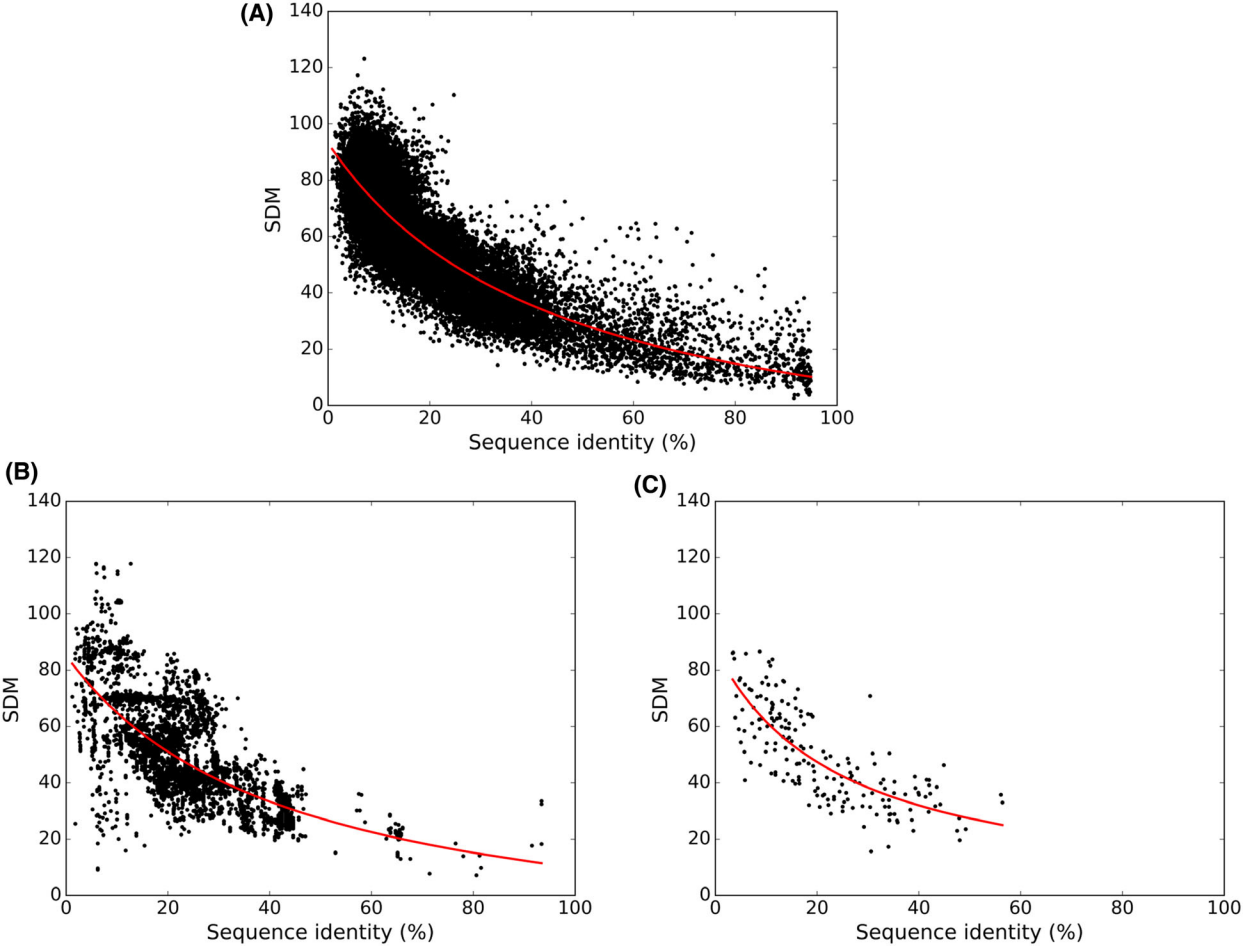
Relationship between sequence similarity and structural deviation. Scatter plot with sequence identity along *X*‐axis and SDM score along *Y*‐axis. (A) Representing non‐interacting homologous pairs of monomeric proteins, (B) Scatter plot of interacting homologous subunits of heterodimers and (C) Scatter plot of interacting homologous domains of two‐domain repeats. The best fit line in red color shows the trend of the distribution.

The datasets comprise of 26,082 pairs (2126 PDB entries) of non‐interacting homologs, 12,639 pairs (1875 PDB entries) corresponding to interacting homologous subunits of heterodimers and 185 entries corresponding to 185 interacting homologous domains from multi‐domain protein structures. The structures in every pair in each of these datasets were superimposed on each other followed by calculating their RMSD, SDM and sequence identity. Figure 1 shows the distribution between sequence identity and SDM score for each of the three datasets. Figure 1A scatter plot which corresponds to monomeric proteins (non‐interacting homologs), that spans all values of sequence identity. Figure 1B,C shows the scatterplots for heterodimers with homologous subunits (interacting homologs) and interacting two‐domain proteins with interaction between homologous domains (interacting homologs), respectively. Overall, only 854 entries out of 12,824 entries retain sequence identity higher than 40% sequence identity. We have 11 such entries in domain repeat‐dataset (11 out of 185 entries) (Fig. 1C) and 843 entries in heterodimers (843 out of 12 639 entries; Fig. 1B). Low frequency of occurrence of domain repeats with high sequence identity has also been noticed in previous studies [2, 3]. Overall, inverse relationship between sequence identity and structural divergence is seen in all the three datasets that was also observed in Chothia‐Lesk analysis for a smaller dataset of non‐interacting homologs. The best fit line highlighted in red color in Fig. 1A–C indicates the trend of the distribution obtained by fitting the data in the equation of (*a*/(*x* + *b*) + *c*). The parameters for three datasets are mentioned in Table 1. This equation was used to capture the hyperbolic inverse relation between SDM and sequence identity.

**Table 1:** The values of the parameters a, b, and c for each of the dataset.

Dataset	a	b	c
Heteromer	4651.99	43.13	−22.61
Domain	2254.55	24.99	−2.71
Non‐interacting	5507.15	44.95	−29.24

**Fig. 2:**
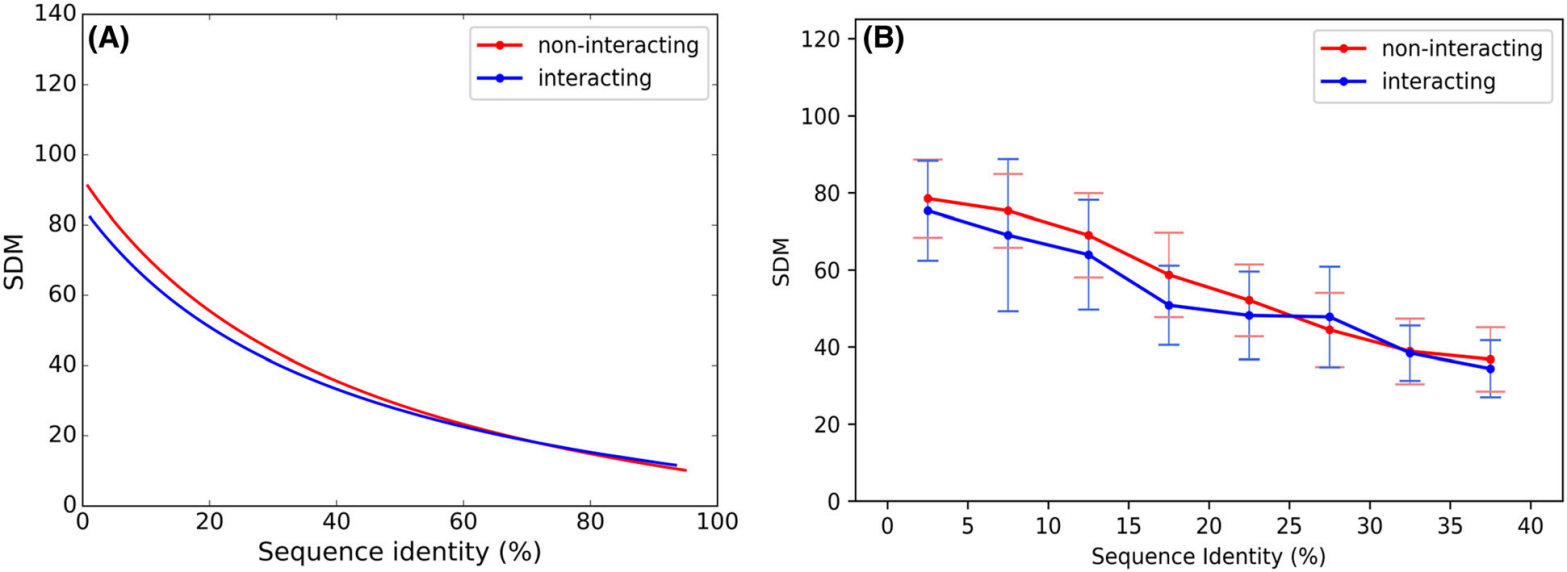
Inverse relationship of sequence similarity and structural difference of interacting and non‐interacting homologous systems. Statistical difference between distributions for non‐interacting and interacting homologous systems with sequence identity along *X*‐axis and SDM score along *Y*‐axis. The red color indicates the non‐interacting homologs and blue color indicates the interacting homologs. (A) Best fit lines representing the trend of data distribution for non‐interacting homologs and interacting homologs. (B) Mean (indicated by dots) and standard deviation (indicated by bars) of SDM scores for every 5% of sequence identity ranging between 0% and 40%.

Owing to sparse data in the two datasets of interacting homologs, we combined the datasets of interacting homologs and compared the distribution with that of non‐interacting homologs. Figure 2A indicates that the best fit line for interacting homologs tend to be lower than the non‐interacting homologs especially at low sequence identity ranges. Further, we compared the two datasets specifically at the low sequence identity range of 0–40%. The data corresponding to 0–40% sequence identity range gives rise to 24,021 numbers of non‐interacting homologous pairs and 4858 numbers of interacting homologous pairs. We calculated the mean and standard deviation of SDM scores for every 5% sequence identity bin, for both non‐interacting and interacting homologs and shown the distribution in Fig. 2B, along with statistical significance provided in Table 2. The difference in the distribution of the points for each interval between the interacting and non‐interacting homologs were checked for using the Kolmogorov–Smirnov test and the difference between the median and the variance were statistically confirmed using Mann–Whitney *U* test. It can be clearly observed that the mean values of SDM scores for interacting homologs is lower than non‐interacting homologs for much of the sequence identity range, especially at the sequence identity less than 25%. This suggests that structural similarity between the interacting homologs is generally higher than the structural similarity between non‐interacting homologs, at sequence identity less than 25%. It was interesting to observe that the interacting domains have higher structural deviation than the non‐interacting domains in the sequence identity range of 25–30%.

**Table 2:** The statistical significance in terms of *P*‐value for every 5% sequence identity range of the SDM score distribution between interacting and non‐interacting homologs.

Sequence identity range (%)	KS‐test (*P*‐value)	Mann–Whitney *U* test (*P*‐value)
≤ 5	7.65E‐05	0.0692
5 < SI ≤ 10	< 2.6E‐16	3.02E‐12
10 < SI ≤ 15	< 2.6E‐16	< 2.6E‐16
15 < SI ≤ 20	< 2.6E‐16	< 2.6E‐16
20 < SI ≤ 25	< 2.6E‐16	< 2.6E‐16
25 < SI ≤ 30	< 2.6E‐16	5.5E‐04
30 < SI ≤ 35	< 2.6E‐16	0.3007
35 < SI ≤ 40	< 2.6E‐16	6.5E‐04

KIn the current work, although the interacting homologs belong to the same fold, they cannot form a perfectly symmetric complex, since the subunits are chemically different owing to low sequence identity between them. This phenomenon of inherent asymmetry was observed by us earlier [22]. We had devised a measure referred as ‘asymmetric score’ that calculates the extent of deviation from perfect symmetry for a pair of subunits in heterodimers, i.e., for a complex generated by crystallographic symmetry axis, the asymmetry score is zero. This method was proposed earlier for homodimers [22]. We have now adapted it for heterodimers, where only the topologically equivalent C‐alpha atoms are considered for calculating asymmetry score to measure global asymmetry. Our earlier studies had shown inherent asymmetry plays a vital role in structure and function of the homodimers [22]. We performed similar study for few cases of heterodimers, case 1: Ku heterodimer (DNA bound [PDB ID: 1JEY] and Unbound [PDB ID: 1JEQ]) and case 2: Endosomal adaptor protein (p14)/MEK‐binding partner 1 (Mp1) [PDB ID: 1VEU] heterodimer. The first case is not part of our dataset but was chosen for the presentation since it interestingly exhibits asymmetry upon DNA binding.

Ku heterodimer (Ku70 and Ku80 subunits) binds to DNA double‐strand breaks and facilitates non‐homologous end joining pathway of DNA repair [23]. The Ku70 and Ku80 subunits share a three domain topology comprising of an amino‐terminal α/β domain, central β‐Barrel domain and a helical C‐terminal region [23]. These three different domain topologies are structurally similar, but collectively contribute to deviation from symmetry due to conformational differences between the subunits. The heterodimeric subunits form a preformed ring‐like structure, encircling the free‐end of the DNA element in a sequence non‐specific manner [23] (Fig. S1A). Comparison of the DNA‐bound form with its unbound form indicates that this complex turns asymmetric upon DNA binding. The unbound form is assigned a gross asymmetry score of 1.93 (Fig. S1B), whereas the DNA‐bound form acquires gross asymmetry score of 3.32 indicating that the asymmetry is required to perform its function.

Endosomal adaptor protein (p14)/MEK‐binding partner 1 (Mp1) heterodimer is an endosomal adaptor/scaffold complex which regulates mitogen‐activated protein kinase (MAPK) signaling. Together, they form a tight dimer (with a Kd of 12.8 nm [24]; Fig. S2A). With an apparent symmetric association, this complex also possess an inherent asymmetry due to difference in their sequences (low sequence identity of 12.5%). The structural asymmetry (gross asymmetry score is 1.64) in a complex is achieved by conformation and orientation of the interacting protein domains. The superposition of p14 and MP1 in (Fig. S2B) shows they are structurally similar (RMSD is 2.60) with conformational differences in the loop regions. The asymmetric loop as highlighted in blue color is functionally important and is required to target p14 to late endosomes [24].

**Fig. 3:**
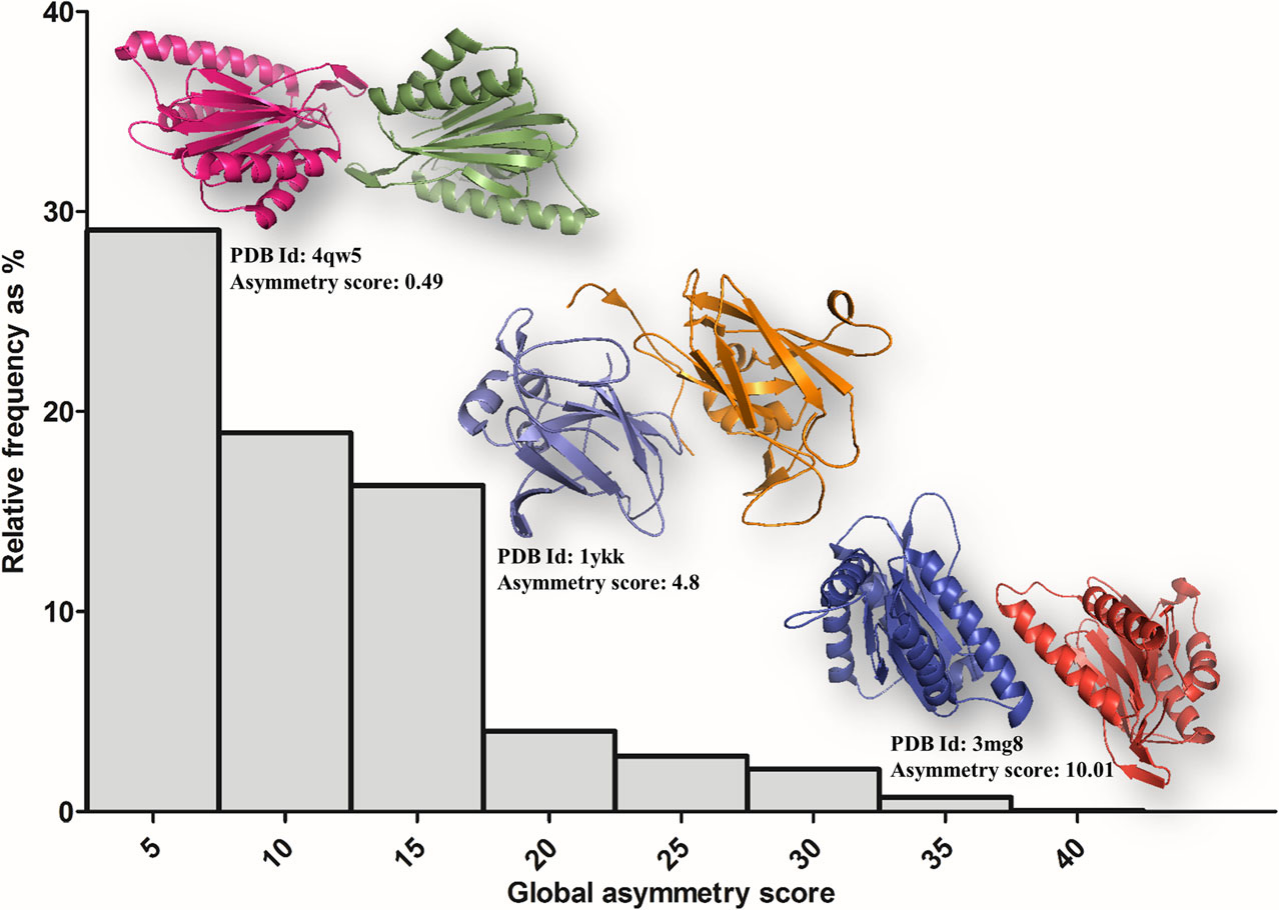
Histogram of asymmetry scores of self‐interacting heterodimers. Values are shown in bins for every 5 Å by bars (along *X*‐axis) and their relative frequency of number of entries (along *Y*‐axis). Three illustrative dimeric structures are shown corresponding to three ranges (PDB codes and asymmetry scores are provided).

Figure 3 shows the distribution of asymmetry scores for the heterodimers used in the dataset, which clearly shows that the frequency distribution is highest for dimers with low asymmetry scores. The inserts in Fig. 3 shows the 3D structures of three heterodimers characterized by different asymmetry scores of 0.49, 4.8 and 10.01 for illustration. These observations indicate preference towards low asymmetry in heterodimers with homologous subunits. One of the requirements for the low asymmetry is high similarity in tertiary structures of the interacting subunits, which were reflected as low SDM scores as well (see previous section).

## Conclusion

Using available 3D structures and analysis of three types of datasets from PDB, we have re‐examined the relationship between sequence identity and structure divergence between homologous proteins, with a commitment to engage in interactions (interacting homologs). As expected, the structural deviation is shown to decrease with increase in sequence identity. The structural deviation between interacting homologs is lower than structural deviation between non‐interacting homologs for a given sequence identity range. This implies that interacting homologs are more similar in their 3D structures than non‐interacting homologs. We also report in this article that such interacting homologs prefer to retain symmetrical association. Therefore, there could be an underlying structural constraint for interacting homologs to retain their overall tertiary structural similarity between them and symmetry in quaternary structure, even at low sequence identity. The inferences drawn from our analyses of remotely related interacting homologs, would hopefully enable future computational methods and approaches for modeling higher order structures and assemblies involving association between homologous chains.

## Conflict of interest

The authors declare no conflict of interest.

## Author contributions

NS and RS conceived the idea for the work. NN and VMP have equal contribution to the work. NN has compiled the manuscript. SV has contributed in formulating the dataset and manuscript preparation.

## Supporting information


**File S1.** Dataset of non‐interacting homologs: Monomeric protein pairs.

**File S2.** Dataset of Interacting homologs: Heterodimers.

**File S3.** Dataset of Interacting homologs: Domain repeats of multi‐domain proteins.

**Fig. S1.** Ku heterodimeric complex. (A) Ku heterodimer (Ku70 and Ku80 subunits) bound to DNA [PDB ID: 1JEY]. (B) DNA‐unbound form of Ku heterodimer [PDB ID: 1JEQ].

**Fig. S2.** p14/MP1 scaffolding complex. (A) p14/MP1 heterodimer [PDB ID: 1VEY], interface residues involved in dimerization are shown in sticks. (B) Superposition of p14 and MP1 protein domains. The loop region highlighted in blue is involved in targeting p14 to late endosome.

## Data Availability

All data generated or analyzed during this study are included as additional files.Data accessibility
