# Supplementary figures and images for: Structures of distantly related interacting protein homologs are less divergent than non‐interacting homologs
Source: FEBS Open Bio. 2022 Oct 17;12(12):2147–53. doi: 10.1002/2211-5463.13492 (PMC9714365; doi:10.1002/2211-5463.13492)

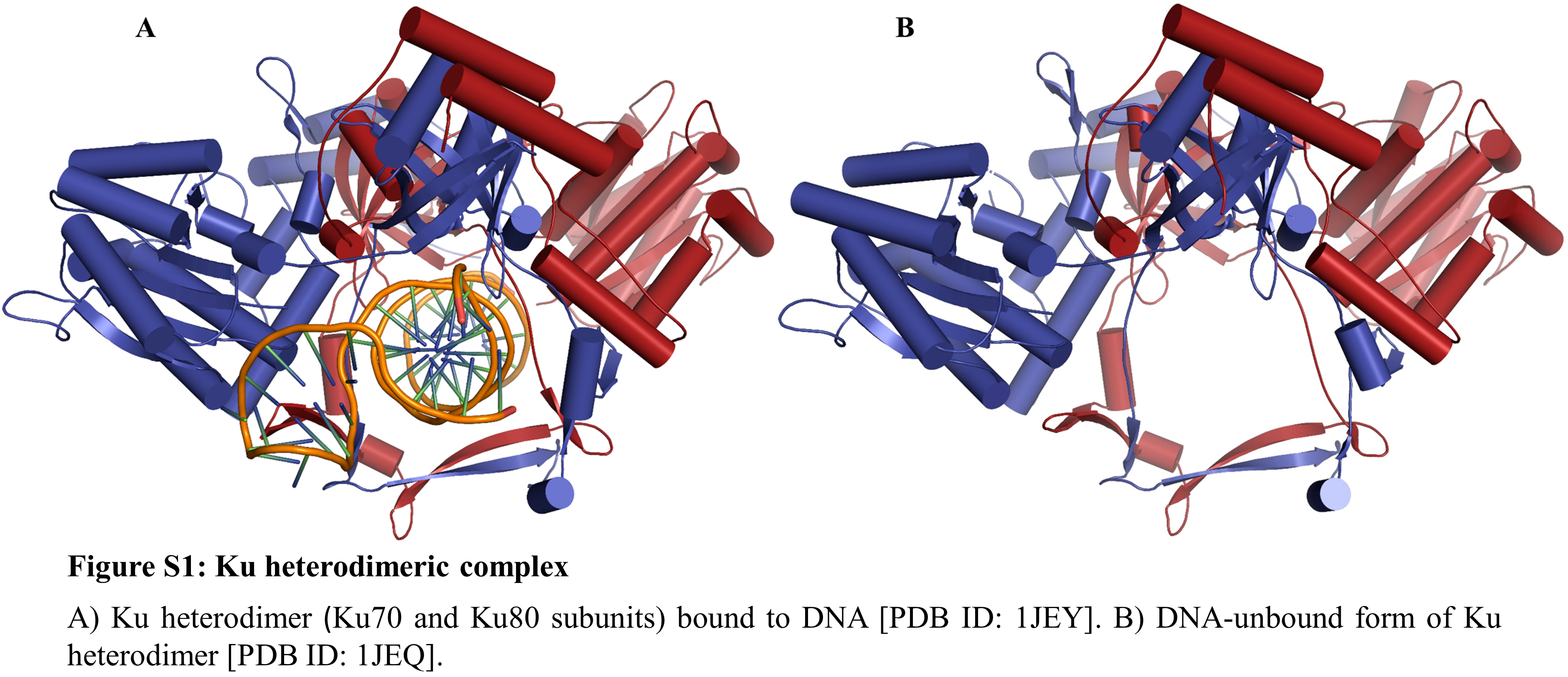

Supplement: Supplementary file 4 — Fig. S1. Ku heterodimeric complex. (A) Ku heterodimer (Ku70 and Ku80 subunits) bound to DNA [PDB ID: 1JEY]. (B) DNA‐unbound form of Ku heterodimer [PDB ID: 1JEQ]. [file FEB4-12-2147-s001.tif]

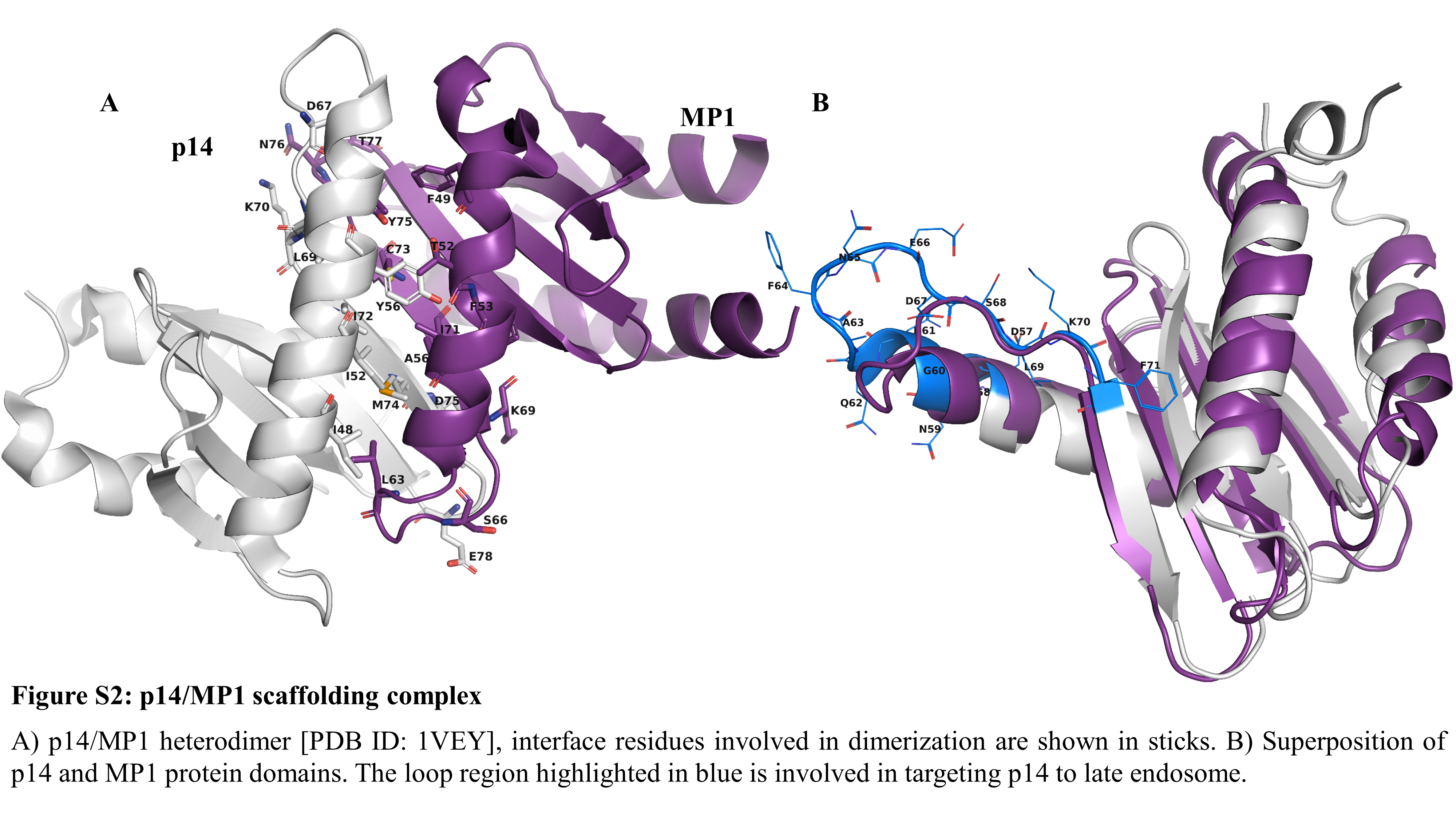

Supplement: Supplementary file 5 — Fig. S2. p14/MP1 scaffolding complex. (A) p14/MP1 heterodimer [PDB ID: 1VEY], interface residues involved in dimerization are shown in sticks. (B) Superposition of p14 and MP1 protein domains. The loop region highlighted in blue is involved in targeting p14 to late endosome. [file FEB4-12-2147-s004.tif]
